# Delamination Detection in Polymeric Ablative Materials Using Pulse-Compression Thermography and Air-Coupled Ultrasound

**DOI:** 10.3390/s19092198

**Published:** 2019-05-13

**Authors:** Stefano Laureti, Muhammad Khalid Rizwan, Hamed Malekmohammadi, Pietro Burrascano, Maurizio Natali, Luigi Torre, Marco Rallini, Ivan Puri, David Hutchins, Marco Ricci

**Affiliations:** 1 Department of Engineering, University of Perugia, Polo Scientifico Didattico di Terni, Strada di Pentima 4, 05100 Terni, Italy; muhammadkhalid.rizwan@unipg.it (M.K.R.); hamed.malekmohammadi@unipg.it (H.M.); pietro.burrascano@unipg.it (P.B.); 2 Department of Civil and Environmental Engineering, University of Perugia, Polo Scientifico Didattico di Terni, Strada di Pentima 4, 05100 Terni, Italy; maurizio.natali@unipg.it (M.N.); luigi.torre@unipg.it (L.T.); marco.rallini@unipg.it (M.R.); ivan.puri@unipg.it (I.P.); 3 School of Engineering, University of Warwick, Library Road, Coventry CV4 7AL, UK; D.A.hutchins@warwick.ac.uk; 4 Department of Informatics, Modeling, Electronics and System Engineering, University of Calabria, 87036 Rende, Italy; m.ricci@dimes.unical.it

**Keywords:** polymeric ablative materials, thermal protection system, thermography, ultrasonic testing, pulse-compression

## Abstract

Ablative materials are used extensively in the aerospace industry for protection against high thermal stresses and temperatures, an example being glass/silicone composites. The extreme conditions faced and the cost-risk related to the production/operating stage of such high-tech materials indicate the importance of detecting any anomaly or defect arising from the manufacturing process. In this paper, two different non-destructive testing techniques, namely active thermography and ultrasonic testing, have been used to detect a delamination in a glass/silicone composite. It is shown that a frequency modulated chirp signal and pulse-compression can successfully be used in active thermography for detecting such a delamination. Moreover, the same type of input signal and post-processing can be used to generate an image using air-coupled ultrasound, and an interesting comparison between the two can be made to further characterise the defect.

## 1. Introduction

Polymeric Ablative Materials (PAMs), also known as Thermal Protection Shielding (TPS) materials, play a strategic role in the aerospace industry. They are used to produce the TPS for aerodynamic surfaces and other structures and are essential during the launch and re-entry phases in a planetary atmosphere [1]. PAMs find also use in liquid-fueled rocket combustion chambers and for providing thermal protection to propulsion devices such as Solid Rocket Motors (SRMs) [2,3]. PAMs can be produced using high char yield thermosets such as phenolics or elastomers such as Ethylene Propylene Diene Monomer (EPDM), Nitrile Butadiene Rubber (NBR), silicone [4,5,6], etc. Different inorganic or organic fillers/fibers are combined with these to increase the ablation and erosion resistance of the final material [2,7].

Elastomeric-based PAMs are mainly used in two fields—in the insulation of SRMs and in the heat shields of space probes and vehicles. SRMs are manufactured from metal or fiber reinforced composite and must be protected against high temperatures (>2800 °C) and high pressures (above 50–60 bar) as a result of combustion gases produced by the propellant. In this case, a sacrificial elastomeric heat shielding liner is placed between the inner surface of the case and the solid fuel grain. In this way, the internal insulation prevents the SRM case from reaching dangerous temperatures. Among the elastomers used for manufacturing SRM liners, EPDM is one of the best matrices as it exhibits low density and an outstanding resistance over weathering effects, oxidation and ozonisation. To increase the heat capacity, thermal stability and fire resistance of the matrix, EPDM is commonly reinforced with high levels of fibrous materials such as glass, carbon and aramid fibers or pulp. High levels of nanosilica are also added to the matrix as they produce a glassy skin, which works as a heat sink and as a self-healing phase once reached the softening temperature. In addition, silicone has also been used with EPDM to produce the insulation liners in different SRM programs [4]. One material based on silicone, DC 93–104, is a highly-loaded silicone rubber filled with SiO_2_, SiC and carbon fibres. DC 93–104 provides high char retention and low ablation rate, even under the influence of high shear stress hyperthermal environments [2].

In the case of space vehicles, the PAM is typically a rigid aeroshell system. Silicone has been used as an infiltrant in different materials, including Acusil® I-IV, SLA-561, SLA-220, and Silicone Impregnated Reusable Ceramic Ablators (SIRCAs) [2,8,9]. However, the payload fairing of the rocket can limit the diameter of the structure. Inflatable Deployable Aeroshells (IDAs) offer an alternative as they are based on flexible materials. Preliminary studies on IDAs indicate that these systems tend to require a smaller payload volume and mass fraction than traditional rigid materials. For example, NASA studied an advanced high temperature IDA for an inflatable re-entry vehicle, where the flexible construction contained high temperature fabrics, insulators, and a gas barrier [9]. Silicone has been used as a liner/coating or as a bonding agent in the production of such materials.

Thus, for both of the above-mentioned applications, the use of silicone enables the production of composite materials having high performance in terms of insulation capability, thermal and erosion resistance. The extreme temperature/pressure conditions that those materials face, and the cost-risk related to the production and operating stage of high-tech aerospace vehicles and components making use of TPS, indicate the importance of detecting any anomaly and defect within them. These defects are likely to arise during the manufacturing stage. The presence of defects such as kissing bonds or de-boding can drastically compromise the capability of a TPS to properly work as an effective thermal barrier. In fact, when a TPS laminate is exposed to hyper-thermal environments characterized by severe heat fluxes and shear stresses—as an example, during a re-entry flight into a planetary atmosphere or inside a SRM—the defects embedded in the structure can promote an increased and uncontrolled loss of material by erosion or spallation [2], substantially reducing the possibility of the TPS to work as a heat sink or, in the worst scenario, producing a catastrophic loss of the item to protect. In other words, due to their intrinsic nature, these defects cannot be considered as controlled voids, i.e., they cannot play the role of voids with a controlled size such as the microballons used to tune the thermal conductivity of syntactic foams. Additionally, the higher the possibility to reduce the loss of charred material via spallation, preserving its role as a heat sink, the higher the ablation efficiency of the TPS material.

Several Non-Destructive Testing (NDT) techniques have been successfully employed for detecting defects/flaws in composite materials often used in the aerospace industry, such as carbon fibre reinforced composite (CFRP), glass laminate aluminium reinforced epoxy (GLARE) and so on. Examples of NDT techniques for investigating composites are eddy-currents [10,11,12,13,14], active thermography [15,16,17,18,19], ultrasonic testing including guided waves [20,21,22,23,24,25], shearography [26,27,28,29], and near infrared spectroscopy [30,31,32,33]. However, the choice of the optimal NDT method for a given sample depends on the physical properties and size of the specimen, as well as whether there is access to one or both sides of the sample.

In this work, a TPS material based on a glass/silicone reinforced laminate was available, which contained an extended area of delamination during manufacture. The mechanical and thermal/electrical properties of the glass fibres, and that of the silicone matrix, suggested that thermography [34] and an air-coupled ultrasound technique (ACUT) [35,36,37] might be proper NDT approaches—thermography is a good technique for thermally insulating materials, and ACUT works better with materials whose acoustic impedance is closer to air; a polymer is a good example of this. The multi-layered and highly-attenuating nature of the material also suggests that techniques would be needed to improve the signal to noise ratio and/or resolution of the imaging. For this reason, a combination of coded waveforms and pulse-compression (PuC) techniques was used to increase the detection capability for a delamination within the sample [38,39,40,41,42,43,44]. Thus, both Pulse-Compression Thermography (PuCT) [41,43] and Pulse-Compression ACUT (PuC-ACUT) [39,43,44] were used and the results compared. In both cases, imaging data is derived from a time analysis of the impulse responses after pulse-compression. Note that PuCT is proposed as the main inspection technique as it can be quickly implemented in situ and can work in reflection mode, meaning that it can be employed quite easily when only a single side of the part to be inspected is accessible. Conversely, PuC-ACUT is a point-by-point technique and it is generally performed in transmission mode requiring access to both sides of the inspected part. Thus, the aerospace industries may not find the latter one as good candidate for a rapid and easy inspection of the TSP state.

The paper is organised as follows: Section 2 briefly overviews the PuC technique and specifically explains how this advanced algorithm can be faithfully exploited and tailored for application in thermography and ACUT. Section 3 describes the materials, experimental setups and methods used to achieve the results showed in Section 4. Finally, conclusions are drawn in Section 5.

## 2. Pulse-Compression 

PuC was firstly introduced in radio detection and ranging (RADAR) to enhance the so-called range resolution while maintaining large inspection ranges [45,46]. Its use later expanded into femtosecond laser applications [47,48,49] and ultrasound [38,39,40]. It has been successfully employed in combination with several NDT methods, such as eddy-currents [50,51], thermography [41,42,52,53], thermography stimulated by eddy-currents [12,54] and vibro-thermography [55]. It has also been used for non-linear measurements [56,57,58]. In this paper, PuC is used for both thermography and air-coupled ultrasound. Although the fundamental aspects of the mathematical theory underlying PuC are the same for both, there are differences in the way that PuC is implemented. 

### 2.1. Pulse-Compression in Air-Coupled Ultrasonic Testing (PuC-ACUT)

In conventional ultrasonic inspection [59], pulsed excitation is used in order to excite the natural bandwidth of the transducer. Assuming a linear response, the system can be modelled as a Linear Time Invariant system from a Dirac Delta-function the δ(t) excitation. If this condition holds, then the impulse response h(t) is obtained from which the presence of defects/anomalies can be inferred, either in the frequency and/or the time domain. Note that the Signal-to-Noise Ratio (SNR) is constrained by the input voltage level as dictated by the available pulser power. To relax this constraint, narrow-band ultrasonic transducers can be employed together with an extended time duration tone-burst to increase the SNR. However, the use of a narrow-band ultrasonic transducer reduces the range resolution, thus decreasing the defect detection capability. PuC, using a coded waveform, can be used both to improve the SNR and to achieve a higher range resolution [60,61], as demonstrated in the inspection of forgings [62], food [63], composites [12,42,64], concrete [60,65] and phononic crystals [66].

PuC relies on the existence of a pair of signals {s(t),Ψ(t)}, such that their convolution $\tilde{\delta }\left(t\right)$ well-approximates the δ(t). This is described in Equation (1), wherein “∗” is convolution operator:

(1) $$s\left(t\right)*\Psi \left(t\right)=\tilde{\delta }\left(t\right)\approx \delta \left(t\right).$$

In Equation (1), s(t) is a coded signal having both time duration *T* and bandwidth *B* limited, and Ψ(t) is the so-called matched-filter. Thus, if s(t) excites the UT transducers and y(t)=h(t)∗s(t) is the output signal collected by an Analog-to-Digital Converter (ADC), an estimate $\tilde{h}\left(t\right)$ of the impulse response h(t) is retrieved by convolving y(t) with Ψ(t). This procedure is carried out for each point *x-y* of the ultrasonic scanned area, thus resulting in a collection of $N_{x}\times N_{y}$ impulse responses $\tilde{h}\left(j_{x},j_{y},t\right)$, where $N_{x},N_{y}$ are the scanning points, respectively, along x, y directions. For simplicity, the PuC process is step-by-step shown in Equation (2) for a single *x*-*y* acquisition point, wherein also the presence of Additive White Gaussian Noise (AWGN) e(t) is considered. e(t) is here assumed as being uncorrelated to the Ψ(t).

(2) $$\tilde{h}\left(t\right)=y\left(t\right)*\Psi \left(t\right)=h\left(t\right)*\underset{=\tilde{\delta }\left(t\right)}{\underbrace{s\left(t\right)*\Psi \left(t\right)}}+\underset{=\tilde{e}\left(t\right)}{\underbrace{e\left(t\right)*\Psi \left(t\right)}}=h\left(t\right)*\tilde{\delta }\left(t\right)+\tilde{e}\left(t\right)\approx h\left(t\right)+\tilde{e}\left(t\right).$$

The SNR gain can be increased by simply increasing *T*, so that the amount of energy delivered to the sample increases, allowing low voltage levels to be used [41,61]. This is of importance in hazardous environments, especially when inspecting highly attenuating material [40]. In the case of PuC-ACUT it is particularly important—air attenuates ultrasonic signals, effectively constraining the technique to frequencies <1 MHz (at which attenuation of air is 120 dB·m^−1^ [67]). 

**Figure 1:**
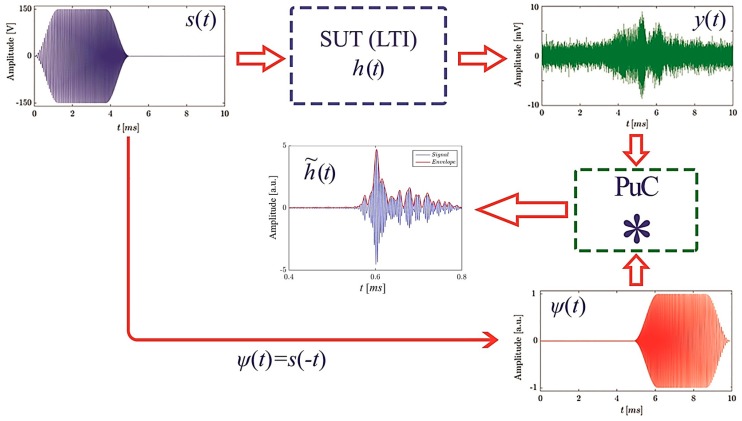
Implementation of the pulse-compression algorithm in air-coupled ultrasonic testing (PuC-ACUT). A coded signal s(t) is input to an ultrasonic transducer, which excites the sample under test (SUT) for a limited time and within a bounded frequency range. The output signal y(t) is then convolved with the matched filter Ψ(t) to obtain an estimate of the sample impulse response $\tilde{h}\left(t\right)$.

However, there are some drawbacks in employing PuC. In particular, *T* and *B* have always limited values whatever the coded excitation used. Thus “side-lobes” are always present in $\tilde{h}\left(t\right)$, and different strategies have been proposed to reduce their influence, based mainly on the use of window functions for smoothing the amplitude of the matched filter and/or the coded input signal, or the use of a tailored Ψ(t) designed from the Wiener filter theory [42,68,69,70,71]. It can be demonstrated that the maximum SNR is obtained when Ψ(t)=s(−t) [72], wherein s(−t) is the time-reversal of the input signal s(t). In this work, a linear frequency modulated “chirp” signal has been employed to excite an air-coupled ultrasonic transducer within its useful bandwidth. Since the aim here is to maximise the detection capability, a Ψ(t)=s(−t) scheme has been adopted. Details of the practical system used for PuC-ACUT are shown schematically in Figure 1, noting that details of the practical experimental design will be given in Section 3. A detailed explanation of the mathematical methods used in PuC is beyond the scope of the present work, but is given in [61], where the “acyclic convolution” scheme adopted here is described.

### 2.2. Pulse-Compression Thermography (PuCT)

In thermography, defects such as delaminations can be inferred from local temperature gradients, which can be detected at the material’s surface by means of a thermal camera [73,74]. The method allows fast inspection and real-time measurements to be carried out over a given detection area by using suitable lenses. IR thermography can be applied both with and without the use of a controlled heat source, leading to active and passive thermography, respectively. In the latter approach, the thermal contrast between defective and non-defective areas is generally low, leading to poor SNRs. Hence, the use of an external controlled heat source is in general beneficial to improve IR thermography performance and detection capability. 

The most common approach is Pulsed-Thermography (PT) [75], which uses high-power flash-lamps (with some kJ of energy) for exciting the sample. The same surface is usually monitored by the thermal camera, i.e., reflection mode. The flash induces a quick change of the SUT’s surface temperature. To retrieve the thermal equilibrium, the heat diffuses toward the inner part of the SUT. Note that in PT, the flash duration is significantly shorter than the typical heat diffusion time duration within the samples, so that the heating is quasi-instantaneous while the cooling is regulated by the thermal characteristics of the SUT, i.e., specific heat, density and thermal conductivity [76,77]. The thermal camera collects a sequence of thermograms during the cooling process and information about the SUT’s surface and inner part can be inferred from the analysis of the cooling trends of each pixel. Precisely, a cooling trend is associated to any pixel $\left(j_{x},j_{y}\right)$ of the IR image, which depends on the pixel emissivity but also on the characteristics of the SUT interior structure. If a thermal anomaly is present, a change in the related pixels’ temperature decay curves can be observed. From a system-theory perspective, the cooling curve of the $\left(j_{x},j_{y}\right)$ pixel as time elapses can be considered as its thermal 1D impulse response $h\left(j_{x},j_{y},t\right)$, wherein the flash excitation is considered as the impulsive Dirac’s Delta excitation δ(t). Again, the LTI system is completely described by its impulse response, thus the SUT is completely characterised by the set of its $\left\{h\left(j_{x},j_{y},t\right)\right\}$, as already stressed for the UT case [42,61]. Therefore, PT is a powerful tool to inspect a huge variety of different samples. However, the major potential drawback in applying PT is that the achieved SNR level is directly related to the flash-lamp power.

In this framework, the use of coded modulated heating stimuli in combination with PuC, i.e., PuCT, is a robust methodology to achieve the desired SNR and still being capable of inferring a high number of features from the impulse response analysis [41,51,52,53,64,69]. In this paper, stimulation for PuCT is provided by LEDs, whose output is modulated by a coded chirp excitation.

In common with the PuC scheme used for ultrasound, Equation (2) is here again exploited to retrieve an estimate of the impulse response $\tilde{h}\left(t\right)$ for each $\left(j_{x},j_{y}\right)$ pixel of the acquired thermal response. As before, the quality of the estimated $\tilde{h}\left(t\right)$ of the real impulse response h(t) obtained by performing PuC depends on the correct choice of both the coded signal parameters *B* and *T* and the related matched filter. Furthermore, in PuCT the retrieved $\tilde{h}\left(t\right)$ quality strictly depends on the correct implementation of the convolution procedure. In fact, issues will arise from the difficulty in realizing a bipolar heating source for thermography. Therefore, an offset must be applied over the coded chirp signal s(t) instantaneous amplitude to drive the employed heating source successfully. It follows that the real employed excitation signal $s_{TR}(t)=s(t)+s_{SQ}(t)$ is the superposition of a chirp signal s(t) and a square pulse $s_{SQ}\left(t\right)=C\left\{\vartheta \left(t\right)-\vartheta \left(t-T\right)\right\}$ where ϑ(t) is the Heaviside step function and T is the input signal time duration. Thus, the true acquired output signal $y_{TR}(t)$ can be described as in Equation (3):

(3) $$y_{TR}\left(t\right)=h\left(t\right)*s\left(t\right)+h\left(t\right)*s_{SQ}\left(t\right)+e\left(t\right)=y\left(t\right)+y_{SQ}\left(t\right)+e\left(t\right)$$

Comparing Equations (2) and (3), it is seen that the contribution of $y_{SQ}\left(t\right)$ from y(t) must be removed before finalizing the PuC algorithm via convolution with the matched filter. Recently, Silipigni et al. [42] proposed a procedure for proper implementing PuC in AT based on extending the $s_{SQ}\left(t\right)$ contribution for some time after T. It was shown that this helps to design an optimized non-linear fitting algorithm, capable of correctly removing the contribution $y_{SQ}\left(t\right)$ from y(t). 

The PuCT procedure uses the following steps:

1. The sample is excited via a chirped heating stimulus of time duration T and with an additional $s_{SQ}\left(t\right)$ contribution for $T_{SQ}=T+T_{h}>T$. Here, $T_{SQ}=110\;s$, T=80 s and $T_{h}=30\;s$. Thus, the sample is kept heated for 30 s after the end of the coded stimulus. Note that $T_{h}$ is the expected time duration of the SUT’s h(t), meaning that $T_{h}$ is the expected cooling time of the sample to retrieve a new equilibrium state or, from a practical point of view, is the duration of interest for the present analysis.
2. Thermograms are acquired for an overall time interval $T_{SQ}$.
3. Apply to the $y_{TR}\left(t\right)$ the step-heating removal procedure (Detrend) for each pixel of the acquired thermogram sequence, thus obtaining y(t). Please refer to Silipigni et al. [42] for more details.
4. A pixel-by-pixel convolution of each y(t) with Ψ(t) is performed, retrieving $\tilde{h}(j_{x},j_{y},t)$ as for Equation (2).

**Figure 2:**
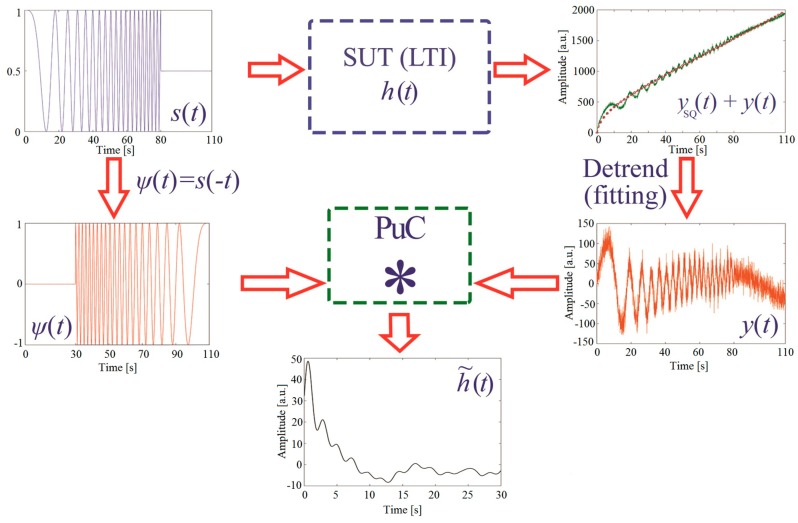
Implementation of the pulse-compression algorithm in active thermography. A coded signal excites s(t) modulates the on/off state of a heat source, exciting the sample under test (SUT) for a limited time and within a bounded frequency range. The output signal $y_{SQ}\left(t\right)+y\left(t\right)$ is de-trend from the step heating contribution $y_{SQ}\left(t\right)$ and y(t) is obtained. This is convolved (“PuC box”) with the matched filter Ψ(t) to obtain an estimate of the sample impulse response $\tilde{h}\left(t\right)$.

The overall procedure is reported in Figure 2, wherein the non-linear fitting is red-marked onto the y(t) plot.

## 3. Materials

### Description of the Sample

**Figure 3:**
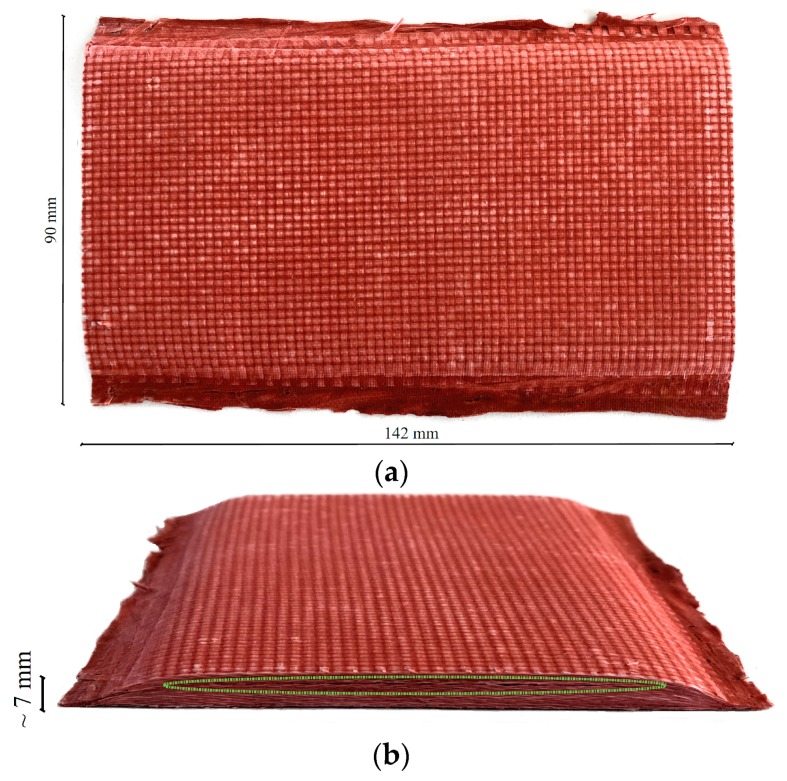
(**a**,**b**) Photographs of the glass/silicone Thermal Protection Shielding (TPS) sample manufactured by Angeloni Tech Materials (Italy). The green dotted line in (**b**) highlights the position of the delamination, which can be barely seen by the naked eye.

A TPS material based on a glass/silicone reinforced laminate was produced with dimensions 142 mm and 90 mm in height and width, respectively, and with a thickness of ~7 mm. A photograph of the TPS sample is shown in Figure 3. A glass fiber plain weave fabric supplied by Angeloni Tech Materials (Italy)—VV 320 P—with nominal areal weight 320 g·m^−2^ was used. The composite was composed by 28 layers stacked to form a balanced symmetric laminate. A high temperature silicone sealing paste formulation was used as an infiltrant (Arexons, MOTORSIL D). However, a silicone/toluene ratio equal to 1:2 was used as a solvent to reduce the viscosity of the silicone paste to an acceptable level. The diluted silicone was stirred to produce a homogeneous infiltrant and then the laminate was produced via hand layup. Once impregnated, the laminate was vacuum-bagged, then cured and consolidated for 24 hours at room temperature. Once un-bagged, the laminate was placed in an oven (80 °C for 2 h) to remove the excess of toluene still present in the composite. The dotted line indicates the delamination introduced during manufacture, which is barely visible to the naked eye from the side view and invisible from both top and bottom surfaces. In addition, the extent of the delamination was unknown and was believed to be caused by a poor content of silicone infiltrant. The thermal diffusivity of the sample was directly measured as 0.18 × 10^−6^ m^2^·s^−1^ by averaging three readings from an Applied Precision ISOMET-2104, whose probe was pressed onto the SUT with a 2 kg force so as to close the delaminated layer. 

## 4. Experimental Setup

### 4.1. PuCT Experimental Arrangement

**Figure 4:**
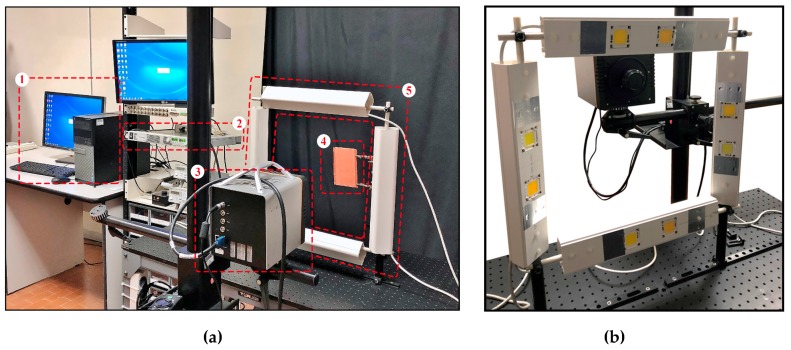
Photographs of (**a**) the complete Pulse-Compression Thermography (PuCT) experimental setup and (**b**) the LED array rotated on its square gantry allowing the active LED elements to be seen. See the main text (Section 4.1.) for details.

A photograph of the experimental PuCT setup is shown in Figure 4a, wherein instruments and materials have been numbered for easiness. (1) A National Instrument PCI-6711 Arbitrary Waveform Generator (AWG) board and a National Instrument 1433 Camera Link Frame Grabber were connected to a PC, and an ad-hoc developed LabVIEW^TM^ virtual instrument managed the signal generation/acquisition. The coded signal was input into a TDK Lambda GEN 750W power supply (2). The AWG board provided both the wanted linear chirp excitation and a reference clock signal (CLK) for triggering the IR camera acquisition, which was a Xenics Onca-MWIR-InSb IR camera (3). The SUT (4) was placed at about 30 cm from the IR camera, and heated-up by a 400W LED chips system (5), which can be better appreciated in Figure 4b. The thermograms were acquired at 40 FPS. The employed chirp s(t) signal had a duration T=80 s and $T_{h}=30\;s$, thus heating up the SUT for 110 seconds overall, within the frequency band of 0–0.5 Hz. 

### 4.2. PuC-ACUT Experimental Arrangement

**Figure 5:**
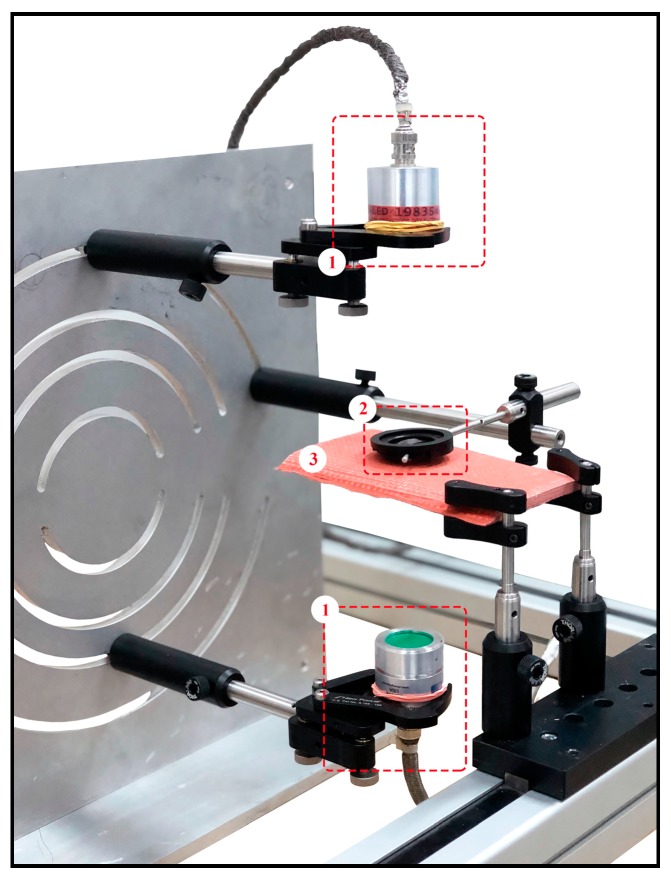
Photograph of the pulse-compression air-coupled ultrasonic setup—(1) transducers, (2) pinhole and (3) sample under test. See the main text (Section 4.2.) for details.

A picture of the PuC-ACUT experimental setup is reported in Figure 5, with all the exploited tools numbered for easy-referencing to the related bracket-numbered items here below. An Arbitrary Function Generator NI-PXI 5412 from National Instruments Inc. provided the wanted chirp excitation between ±3 V range on a load of 50 Ω, which was then amplified to ±150 V by a WMA-300 from Falco System. (1) A pair of air-coupled focused transducers (focal length equal to 100 mm) from The Ultran Group (Boston, USA), having nominal central frequency of 200 kHz and aligned on-axis in air at 200 mm one from the other, was employed for the signal generation/acquisition. Note that an acoustic pinhole (2) with a nominal circular aperture of 2 mm was placed at the focal length distance just above the SUT (3) to further reduce the acoustic beam size. The receiving transducer, i.e., the bottom one, was directly digitalized by an ADC NI-PXI 5105 with 12 bit of resolution at a sampling rate of 4 MS·s^−1^. The SUT was hold in place and connected to a 2D motorized stage plotter from LAM Technologies (Sesto Fiorentino, Italy) by two clampers. The investigated TPS material was placed exactly in the middle between the transducers. The employed chirp signal had a duration T=4 ms, linearly-sweeping within the frequency range 125–375 kHz.

## 5. Results

**Figure 6:**
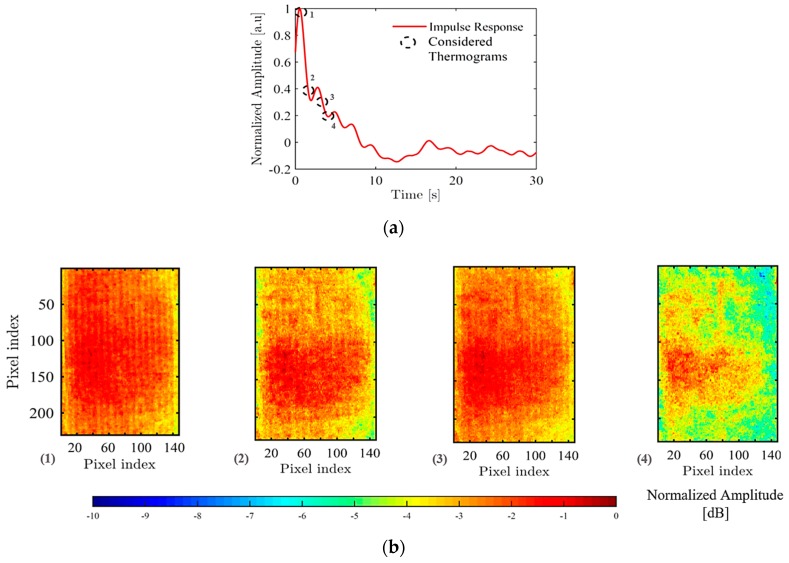
(**a**) Normalised estimated impulse response $\tilde{h}\left(t\right)$ for a single pixel of the reconstructed thermograms after pulse-compression. (**b**) A series of thermograms obtained by imaging pixelwise the $\tilde{h}\left(t\right)$ amplitude (normalised and in dB scale) as time elapses, i.e., at 1, 2, 3 and 4 s. An extended area of higher temperature is noticed, which is likely to be related to the delamination.

An estimate of the impulse responses {$\tilde{h}(j_{x},j_{y},t)$} was obtained after PuCT for the whole captured thermogram series, following the procedure detailed in Section 2. An example of a typical $\tilde{h}(t)$ is shown in the top part of Figure 6 for a single pixel of the acquired IR data, whose amplitude has been normalised to its maximum. A sidelobe-affected cooling trend is noticed after the peak rise in temperature/emissivity occurring at around 1 s. This is mainly due to the chosen chirp coded excitation and matched filter. Nevertheless, meaningful IR images of the SUT can be obtained by plotting the maximum amplitude of the acquired $\tilde{h}(t)$ at different time instants for each pixel. Four different time delays after excitation (1, 2, 3 and 4 s) were analysed, and the obtained images are depicted in Figure 6a in a normalised dB scale. The four time values are marked as black-dotted circles in the $\tilde{h}(t)$ plot. It can be seen that an extended area of the surface showed higher temperature/emissivity with respect to the surrounding one, and this contribution gets lower and lower in amplitude as time elapses, i.e., as the heat diffuses within the SUT and a new thermal equilibrium state is achieved. This “hot” area is likely to be related to the presence of an extended delamination, which seemed to affect almost the whole SUT’s main axis, leaving unaltered just a small portion of it at its bottom end. 

**Figure 7:**
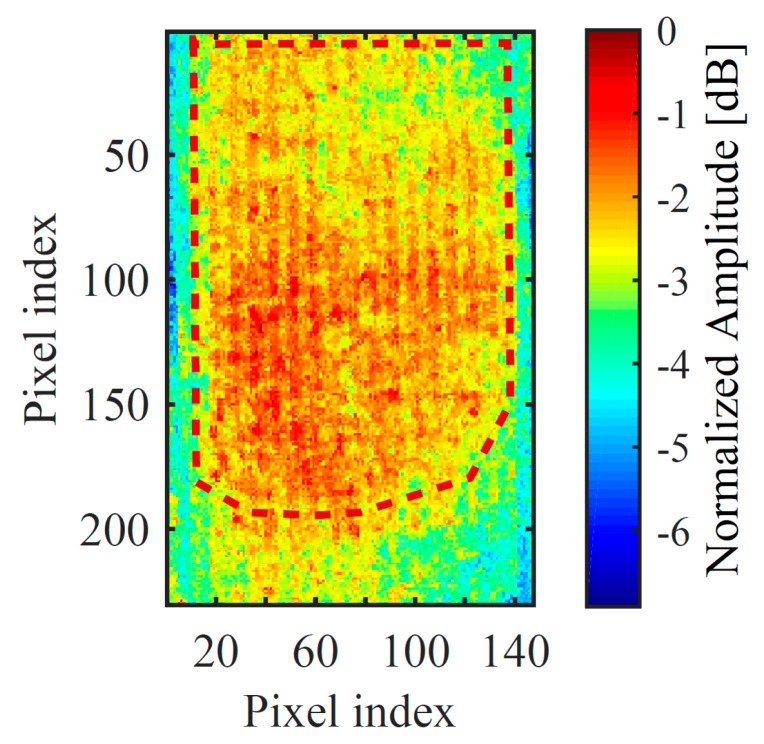
First Principal Component reconstructed image after pulse-compression. The potential delaminated area is showed by the “hot” pixels area, and it was marked with red dots to be easily identified.

Principal Component Analysis (PCA) was used to enhance the visibility of the delaminated area [78]. Figure 7 shows the first PCA component in normalised dB scale—the considered delaminated area has been marked on it. This confirmed the presence of an extended delaminated area within the sample.

**Figure 8:**
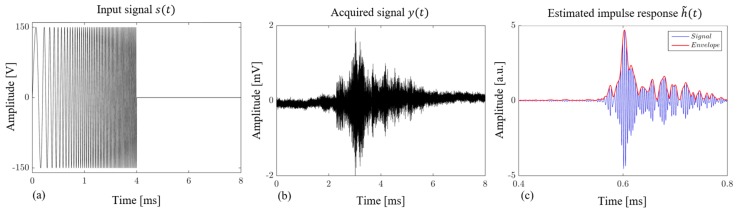
(**a**) Amplified chirp input signal and (**b**) acquired data from a single scanning step onto the delaminated part. (**c**) Obtained impulse response after pulse-compression, together with its envelope.

Consider now the ultrasonic PuC-ACUT measurement, which was employed to verify and corroborate the obtained PuCT results and further sizing of the delamination. Figure 8a depicts the driving chirp signal after being amplified, whilst an example of a signal acquired over a delaminated area is depicted in Figure 8b. Note that although the attenuation is quite high, a genuine signal is still being obtained after PuC, i.e., $\tilde{h}(t)$. This is shown in Figure 8c together with its envelope obtained after Hilbert transform. 

**Figure 9:**
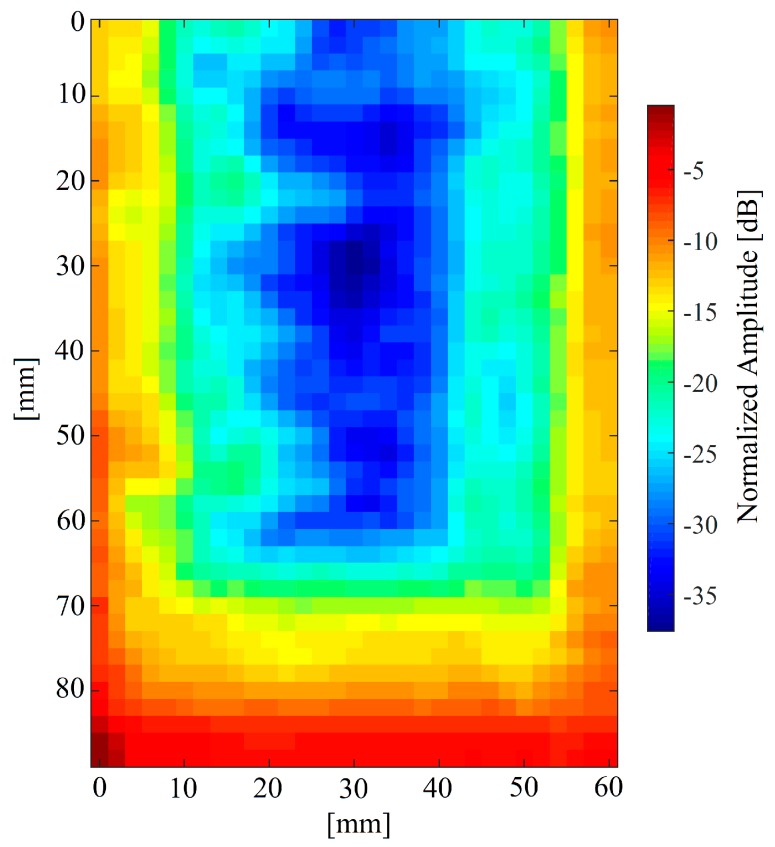
Pulse-compression air coupled ultrasonic results. The image shows the $\tilde{h}\left(t\right)$s normalised amplitude (dB) and the presence of an extended delamination is visible as pixel area with higher attenuation.

A C-Scan can be obtained by showing $\tilde{h}(t)$’s maximum value within 0.5–0.7 ms range for each acquisition point. Thus, a collection of $\tilde{h}(t)$s was obtained by moving the sample in 2 mm intervals using the motorised scanning stage and acquiring a signal after each movement. An area of 90 mm by 60 mm over the sample was scanned, this being restricted by the mechanical scanning arrangement. This area partially covered the delaminated area along the SUT’s main axis and the whole sound part of the sample, as indicated by the PuCT results shown earlier in Figure 7. The obtained scan is presented in Figure 9. 

**Figure 10:**
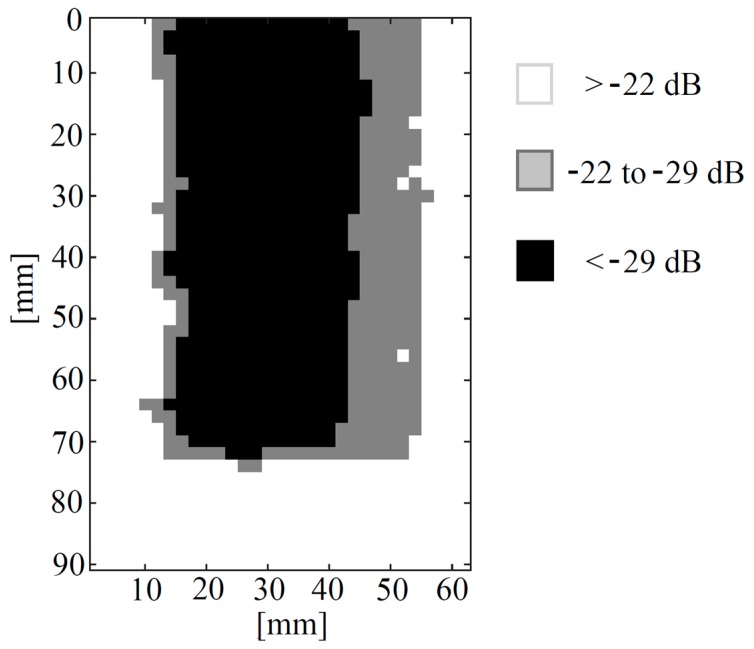
Pulse-compression air coupled ultrasonic results. The image shows the $\tilde{h}\left(t\right)$s normalised amplitude (dB) but with three different bounded amplitude levels.

As expected, ultrasonic attenuation is greatest within the central part of the TPS sample, indicating an extended delamination, leaving unaltered only a small part at its bottom end and surrounding it. It can be noted that the retrieved amplitudes tend to cluster within well-defined areas. Figure 10 shows an image wherein −22 dB and −29 dB amplitude levels of thresholding were used. The results in Figure 10 are in good agreement with those showed in Figure 7, as an extended delaminated area was found for both the employed techniques, which leaves just a small portion of the sample at its bottom end unaltered. Further, Figure 9 and Figure 10 show that the delamination is likely to be not-uniform within the sample, meaning that the delamination gets weaker when approaching toward the edges of the sample and towards its lower end. In other words, a smooth transition between sound and delaminated areas is thought to be present within the sample, leading to a “U-shaped” defect. 

## 6. Conclusions

A glass/silicone polymeric ablative material, employed as thermal protection systems in the aerospace industries was inspected with both active thermography and air-coupled ultrasonic testing. The manufactured sample contained a delamination, which arose during the manufacturing stage, a fact that can lead to catastrophic failure if not detected properly in situ. The sample characteristics, i.e., its multi-layered nature and its usage (where only one side is thought to be generally accessible for inspection), suggested that the combined use of coded signals and pulse-compression could help to detect the delamination in active thermography. The same pulse-compression procedure was used in air-coupled ultrasonic testing to corroborate the thermal results, even if in this case the measurements were performed in through-transmission mode. It can be concluded that pulse-compression thermography, using low power and cheap LED chips, was able to detect the delaminated area within the glass/silicone TPS material, whereby a more precise delamination sizing was achieved by using principal component analysis. Pulse-compression air-coupled ultrasonic testing confirmed the presence of an extended delamination and helped to get a more precise idea of the delamination magnitude. In both cases, it has been demonstrated that pulse-compression is a powerful tool to inspect highly-attenuating material and to enhance the signal-to-noise ratio.

It should be noted that while active thermography turned out to be a rapid and reliable method for inspecting glass/silicone material in reflection mode, thus being a good candidate for the in-situ inspection of aerospace component, the results achieved with this method suffered from lateral diffusion. This hampered the faithful sizing of the delamination with respect to the ultrasonic technique (please compare Figure 7 with Figure 9 and Figure 10). In other words, the delamination was better characterised using air-coupled pulse-compression ultrasonic testing. Future work will try to combine the advantage of advanced reconstruction algorithms in pulse-compression thermography, such as the one reported in [79], which could aid in achieving a better defect sizing.
